# Variations in area-level disadvantage of Australian registered fitness trainers usual training locations

**DOI:** 10.1186/s12889-016-3250-3

**Published:** 2016-07-11

**Authors:** Jason A. Bennie, Lukar E. Thornton, Jannique G. Z. van Uffelen, Lauren K. Banting, Stuart J. H. Biddle

**Affiliations:** Active Living and Public Health Program, Institute of Sport, Exercise and Active Living (ISEAL), Victoria University, Melbourne, VIC Australia; Centre for Physical Activity and Nutrition Research, School of Exercise and Nutrition Sciences, Deakin University, Melbourne, VIC Australia

**Keywords:** Personal trainers, Socioeconomic disadvantage, Strength training, Aerobic physical activity

## Abstract

**Background:**

Leisure-time physical activity and strength training participation levels are low and socioeconomically distributed. Fitness trainers (e.g. gym/group instructors) may have a role in increasing these participation levels. However, it is not known whether the training location and characteristics of Australian fitness trainers vary between areas that differ in socioeconomic status.

**Methods:**

In 2014, a sample of 1,189 Australian trainers completed an online survey with questions about personal and fitness industry-related characteristics (e.g. qualifications, setting, and experience) and postcode of their usual training location. The Australian Bureau of Statistics ‘Index of Relative Socioeconomic Disadvantage’ (IRSD) was matched to training location and used to assess where fitness professionals trained and whether their experience, qualification level and delivery methods differed by area-level disadvantage. Linear regression analysis was used to examine the relationship between IRSD score and selected characteristics adjusting for covariates (e.g. sex, age).

**Results:**

Overall, 47 % of respondents worked in areas within the three least-disadvantaged deciles. In contrast, only 14.8 % worked in the three most-disadvantaged deciles. In adjusted regression models, fitness industry qualification was positively associated with a higher IRSD score (i.e. working in the least-disadvantaged areas) (Cert III: ref; Cert IV β:13.44 [95 % CI 3.86-23.02]; Diploma β:15.77 [95 % CI: 2.17-29.37]; Undergraduate β:23.14 [95 % CI: 9.41-36.86]).

**Conclusions:**

Fewer Australian fitness trainers work in areas with high levels of socioeconomic disadvantaged areas than in areas with low levels of disadvantage. A higher level of fitness industry qualifications was associated with working in areas with lower levels of disadvantage. Future research should explore the effectiveness of providing incentives that encourage more fitness trainers and those with higher qualifications to work in more socioeconomically disadvantaged areas.

**Electronic supplementary material:**

The online version of this article (doi:10.1186/s12889-016-3250-3) contains supplementary material, which is available to authorized users.

## Background

Chronic disease prevention is a leading global public health issue [1]. There is strong evidence that a lack of sufficient physical activity is independently associated with an increased risk of several major chronic diseases including coronary heart disease, type 2 diabetes, colon and breast cancer, depression, and Alzheimer’s disease as well as all-cause mortality [1, 2]. For the prevention of chronic diseases, The World Health Organization (WHO) recommends that adults participate in (i) at least 150 min/week of moderate (e.g. walking) or 75 min/week of vigorous-intensity aerobic physical activity (e.g. jogging), or an equivalent combination of both, and (ii) 2 or more days per week of muscle strengthening activity involving major muscle groups [3]. In addition, other health organisations, such as the American College of Sports Medicine, recommend that adults should engage in specialised exercises to enhance neuromotor fitness, (e.g. coordination, agility and balance) by doing exercises on unstable surfaces, such as balance beams or wobble boards and flexibility-related activities (e.g. passive and active stretching, tai chi, yoga) [4]. While the current evidence base is limited to support the health benefits of these activity modes among apparently healthy adults, engagement in neuromotor fitness and flexibility training are likely to be beneficial for older adults at risk of falling.

Despite of the promotion of existing physical activity recommendations, population adherence remains low. Recent estimates based on self-reported data suggest that globally between 40 and 60 % of adults meet the moderate to vigorous-intensity aerobic physical activity guidelines [5], 15–30 % meet the strength training guidelines [6–10], whilst only 10–20 % meet the combined moderate to vigorous-intensity aerobic physical activity-strength training guidelines [6, 11, 12]. Given these low levels, physical activity adherence is considered one of the biggest challenges in health promotion [13].

It has been recently proposed that fitness trainers, such as personal trainers, gym or group instructors, have a potentially important and underutilised role in promoting and supporting physical activity and exercise [14, 15]. Qualified fitness trainers should be trained in the principles of exercise prescription, such as pre-screening, goal setting, assessment and monitoring and program design [16]. Moreover, fitness trainers have access to exercise equipment to deliver a wide range of exercise modalities (e.g. stationary bikes, strength training equipment, stability balls). However, the effectiveness of fitness trainers in reaching the most inactive populations remains unknown. Research into factors associated with physical activity shows that those who experience socioeconomic disadvantage are consistently among the most inactive population subgroups [17]. Encouragement to engage in physical activity may be more limited amongst socioeconomically disadvantaged individuals since the engagement of a fitness trainer is contingent upon the ability to afford this service.

Another potential factor limiting engagement with fitness trainers may be a lack of availability of professionals within an individual’s immediate environment, such as a neighbourhood. This is consistent with the emerging research describing the role of area-level disadvantage on physical activity levels [18]. In brief, after controlling for individual factors (e.g. age, gender, health-status), low physical activity levels observed among disadvantaged populations are partly explained by several area-level factors including real and perceived access to recreation facilities [18, 19].

Fitness trainers work in a variety of indoor settings (e.g. large fitness centres, health clubs, small studios) and outdoor settings (e.g. local parks, recreation reserves) [20]. At present, research on access to exercise facilities have mostly examined structured (e.g. gyms, health clubs, outdoor exercise stations) [21, 22] and unstructured exercise facilities (e.g. parks) [23]. Studies have shown that fitness centre density are distributed by area-level disadvantage, with more advantaged areas having more facilities [21]. While these studies provide insights into the distribution of exercise facilities, little is known on where the services provided by fitness trainers are currently distributed within the community.

In 2011, it was estimated that ~30,000 adults in Australia were employed full-time, part-time or casually as fitness trainers [24], highlighting a great potential for a wide reach of fitness service provision. Fitness trainers are simply a service provided for community members to help them engage in correctly monitored physical activities. Whilst individuals can maintain fitness simply through the presence of a path (which they can walk or jog on) there are many other facets of the environment that can lead to greater participation in physical activity. Local provision of fitness trainers may be one such factor that to date has not been explored with regards to location.

Using a large sample of Australian fitness trainers, the primary aim of this study was to examine if training locations (e.g. large fitness centres, small studios, local parks) are distributed by area-level disadvantage. A secondary aim was to examine whether characteristics of trainers (e.g. qualifications, years of experience) were associated with area-level disadvantage.

## Methods

### Recruitment

The study protocol was approved by the Victoria University Ethics Committee (Ref: HRE 14–070) and informed consent was obtained from each participant. Our sample of currently registered Australian fitness trainers was recruited via an online survey. To aid with recruitment, we collaborated with Fitness Australia, a not-for-profit, member-based industry association representing Australian fitness trainers. Fitness Australia classifies a fitness trainer as someone who holds a minimum Certificate III in Fitness or a completed Human Movement/Exercise Science Degree [20].

In June 2014, an email was sent inviting all registered fitness trainers within Fitness Australia’s database to complete an online survey. Two reminder emails were sent during the following four weeks. In total 9,100 emails were successfully delivered to registered fitness trainers. Of those who received the email, 1,980 opened the online survey and 1,189 completed the entire survey (response rate = 13.1 %).

### Measures

#### Levels of socioeconomic disadvantaged within the usual training location

To determine the postcode of where the fitness trainers provide their usual services, we asked the following question: *‘What is the post code of the fitness industry setting you commonly work in?’ (If you work in more than one setting, please choose the ONE in which you spend most of your time working in as a fitness professional).* To determine the levels of socioeconomic disadvantage in the postcode of respondents’ usual training location, we used the Australian Bureau of Statistics (ABS) Socio-Economic Indexes for Areas (SEIFA) Index of Relative Socioeconomic Disadvantage (IRSD) [25]. In brief, an IRSD score is assigned to areas and indicates the collective socioeconomic characteristics of the people living in an area, with key indicators including education, employment status, marital status, vehicle ownership, and income [25]. The IRSD has previously been used as a standardised measure of area-level disadvantage in Australian studies examining physical activity [18] and nutrition-related behaviours [26]. A lower IRSD score indicates relatively greater disadvantage [25]. IRSD scores for the postcode were matched to the postcode of the training location reported by the fitness trainer.

#### Characteristics of fitness trainers

Respondents were asked to report how many years they had worked as a fitness trainers, their qualification, and the mode (i.e. personal training, group training) and setting of their services (e.g. large gym, outdoor setting). See Table 1 for detailed response categories. Each of these factors are plausibly linked to the quality and price of the service delivered [27], and therefore have relevance to socioeconomic inequalities.

**Table 1:** Sociodemographic and fitness industry-related characteristics of a sample of Australian fitness trainers

	Fitness trainers
	*n* = 1,189
	Mean (SD)
Age	39.3 (11.5)
Sex	n (%)
Males	343 (28.8)
Females	846 (71.2)
Region	
Urban	788 (66.3)
Regional/remote	401 (33.7)
Time as a fitness industry professional	
Less than 12 months	187 (15.7)
1–3 years	354 (29.8)
4–9 years	219 (18.4)
10 years or more	429 (36.1)
Fitness industry qualification	
Certificate III in Fitness	156 (13.2)
Certificate IV in Fitness	806 (67.8)
Diploma of Fitness	110 (9.2)
Undergraduate, graduate or postgraduate degree in Exercise Sciences	111 (9.4)
Mode of training offered	
*Personal trainer*	
No	561 (47.2)
Yes	628 (52.8)
*Group Instructor*	
No	716 (60.2)
*Yes*	473 (39.8)
Fitness industry setting	
Large corporate gym or community health centre (>500 members)	396 (33.3)
Medium size gym or fitness centre (between 100–500 members)	213 (17.9)
Small studio setting	178 (15.0)
Outdoor setting	192 (16.2)
Train clients from own home	100 (8.4)
Visit client’s homes	49 (4.1)
Other	61 (5.1)

#### Covariates

The survey included questions about general demographic factors including sex, age, and region of residence collapsed into: (i) urban (capital cities or metropolitan centres with a population of >100,000); (ii) regional/(inclusive of both large rural centres with a population between 5,000 and 99,000); and (iii) remote (areas with a population of <5,000) [28].

### Statistical analysis

Descriptive statistics were used to describe the demographic profile of respondents and distribution training location by IRSD score. In the analytical models, linear regression were used to examine the relationship between characteristics of the fitness professional and levels of disadvantage in the postcode of the training location, adjusting for covariates. Consistent with previous studies [29], the regression model was run for the continuous SEIFA IRSD score (centred on mean) and for the deciles of SEIFA IRSD (which were also modelled as continuous). The online survey was constructed in a manner that eliminated missing data. All analyses were undertaken using Stata 12.1.

## Results

A total of 1,189 fitness trainers completed the online survey. As shown in Table 1, the sample comprised a greater proportion of females (71 %) and those from urban settings (66 %). The mean age of participants was 39.3 years (±11.5). Over three quarters had a either a Certificate III or IV in Fitness, and over half were personal trainers. Over one third worked in a large corporate gym or community health centre and had been working in the fitness industry for 10 years or more (Table 1).

### Representativeness of the sample

To examine the representativeness of the sample, we made comparisons with existing data on Australian fitness trainers (Additional file 1: Table S1). When comparing the data to the Australian Bureau of Statistics ‘Employment in Sport and Recreation Report 2011’, our study oversampled for females (ABS 2011 = 66 %, vs current study =71 %). Based on Fitness Australia’s publicly available ‘*2012 Fitness Industry Workforce Report*’ [20], we oversampled for older professionals (proportion aged ≥40 years; Fitness Industry Workforce Report = 28.8 %, vs current study = 48.6 %). Comparisons to the industry report show that our sample had similar proportions of trainers by fitness industry qualifications and educational levels and located across Australian States or Territories [20].

### Distribution of training locations by area-level disadvantage

**Fig. 1:**
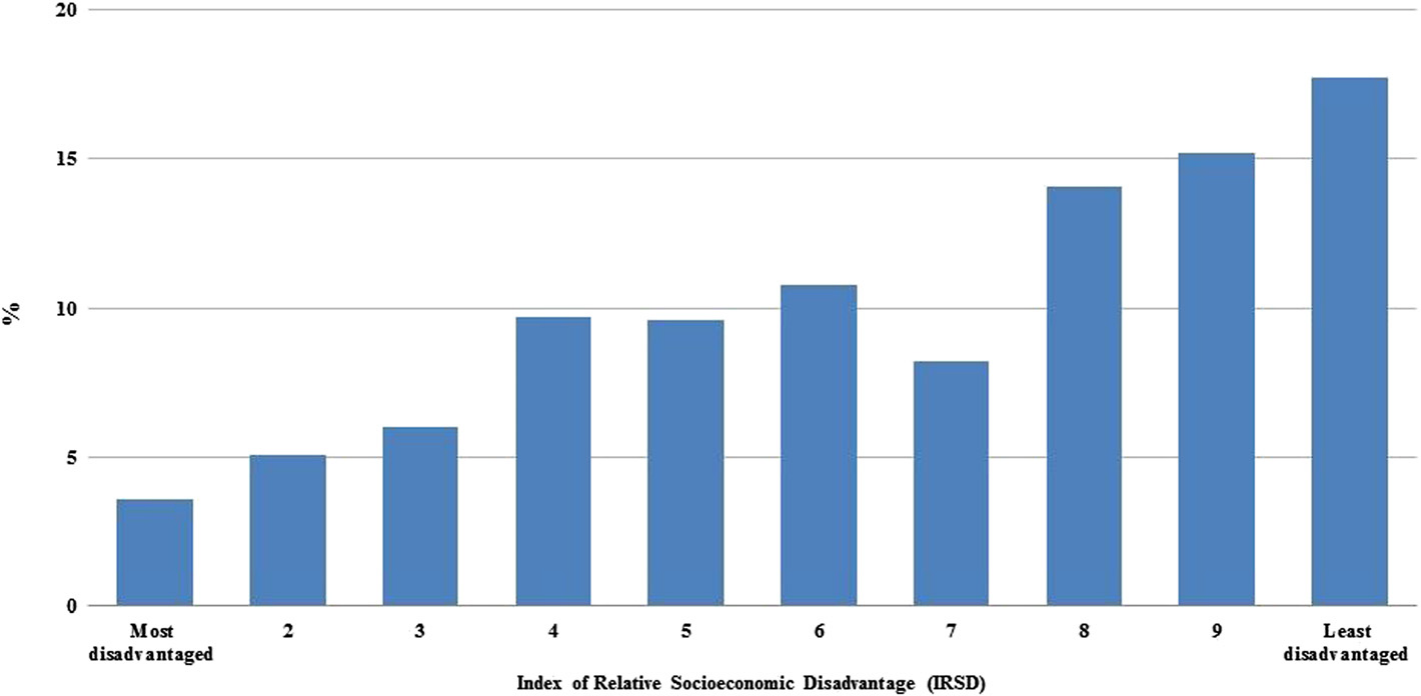
Percentages (%) of fitness trainers (*n* = 1,189) in each decile of socioeconomic disadvantage

The proportion of registered fitness trainers working in each decile of socioeconomic disadvantage is shown in Fig. 1. There was a noticeable trend for a larger proportion of trainers to work in areas with lower levels of disadvantage. Almost five times more trainers worked in the least disadvantaged areas compared to the most disadvantaged areas (17.7 % versus 3.6 %, respectively). Almost half (47 %) of the fitness trainers sampled trained in the three areas that were the least disadvantaged. In contrast, only 14.8 % worked in three most disadvantaged areas (Fig. 1).

### Fitness trainer’s characteristics by area-level disadvantage

As shown in Table 2, area-level disadvantage of the fitness trainer’s training location was not associated with experience and the training setting. Having higher qualifications, however, was positively associated with IRSD score and decile ranking (both indicative of lower levels of socioeconomic disadvantage). Compared to those who had a Certificate III in Fitness, those with Certificate IV in Fitness (ß 13.4 95 % CI: 3.86-23.02), a Diploma of fitness (ß 15.77, 95 % CI: 2.17-29.37), or an Undergraduate degree (ß 23.14, 95 % CI: 9.41-36.86) each trained in areas with a a higher IRSD score indicating lower level of disadvantage (coef. for continuous IRSD score presented in text; positive associations also found for decile measure). There was a weak association suggesting that conducting group training was associated with lower IRSD (i.e. greater disadvantage) (ß −0.36; 95 % CI −0.64, −0.07).

**Table 2:** Adjusted^a^ linear regression analysis and 95 % confidence intervals (95 % CI) for associations between explanatory factors and the Index of Relative Socioeconomic Disadvantage (IRSD) of fitness trainers usual training location Australian fitness trainers (*n* = 1,189)

	*IRSD continuous*		*IRSD deciles (treated as continuous)*	
	Adjusted		Adjusted	
	Coef (95 % CI)	*p* value	Coef (95 % CI)	*p* value
Time as a fitness trainer				
Less than 12 months	-		-	
1–3 years	9.31 (−0.60, 19.22)	0.066	0.42 (−0.01, 0.85)	0.056
4–9 years	8.68 (−2.36, 19.72)	0.123	0.31 (−0.17, 0.79)	0.209
10 years or more	6.88 (−3.38, 17.13)	0.189	0.30 (−0.15, 0.74)	0.189
Fitness industry qualification				
Certificate III in Fitness	-		-	
Certificate IV in Fitness	13.44 (3.86, 23.02)	0.006	0.60 (0.20, 1.02)	0.004
Diploma of Fitness	15.77 (2.17, 29.37)	0.023	0.64 (0.05, 1.22)	0.034
Undergraduate, graduate or postgraduate degree in Exercise Sciences	23.14 (9.41, 36.86)	0.001	1.10 (0.51, 1.69)	<0.001
Mode of training offered				
Personal trainer				
No	-		-	
Yes	5.61 (−0.75, 11.98)	0.084	0.26 (−0.02, 0.54)	0.065
Group Instructor				
No	-		-	
Yes	−6.34 (−12.87, 0.20)	0.057	−0.36 (−0.64, −0.07)	0.014
Fitness industry setting				
Large corporate gym or community health centre (>500 members)	-		-	
Medium size gym or fitness centre (between 100–500 members)	−1.40 (−10.78, 7.98)	0.770	−0.06 (−0.46, 0.34)	0.785
Small studio setting	3.82 (−6.10, 13.72)	0.450	0.17 (−0.26, 0.60)	0.431
Outdoor setting	2.88 (−6.71, 12.48)	0.555	0.14 (−0.2, 0.56)	0.503
Train clients from own home	−4.26 (−16.54, 8.01)	0.496	−0.20 (−0.73, 0.33)	0.467
Visit client’s homes	2.81 (−13.84, 19.45)	0.741	0.25 (−0.47, 0.97)	0.494
Other	−8.38 (−23.64, 6.89)	0.282	−0.39 (−1.05, 0.28)	0.253

^a^All models adjusted for sex, age, and region of residence

## Discussion

To our knowledge, this study is the first to describe Australian fitness trainer’s usual training locations and characteristics of trainers by area-disadvantage. Findings revealed areas with high levels of socioeconomic disadvantage had fewer and lower qualified fitness industry professionals training within them.

Previous research from the UK, US and Europe has shown a comparable gradient in area-level disadvantage for aspects of the built environment related to physical activity, such as distribution of fitness centres and outdoor exercise facilities [19, 21–23]. However, our study is the first to explore fitness trainer’s usual training locations. This is an important service thus far overlooked. While it is only possible to speculate on the causes of the gradient we observed, it may be due to market force and economic factors. Fitness trainers choose to work in areas in which they encounter clients who pay for their services. It may also be because facilities such as fitness centres and open space less prominent and of lower quality in the most disadvantaged areas limiting the locations where training could be undertaken. Further research is required to determine the reasons behind the observed socioeconomic patterning. Nonetheless, from a public health and social inequality perspective, a lack of fitness trainers currently working in the most disadvantage areas warrants attention.

Our finding that higher qualified fitness trainers were less likely to work in areas of greater disadvantage also deserves consideration. It is plausible that higher educated fitness trainers may charge higher fees for services which in turn affect their choice of location. Further research needs to determine whether a higher level qualification is related to better delivery of fitness services. However, trainers with university qualifications are educated on specific exercise treatment plans for managing and reducing the risk of many chronic illnesses including neurological and neuromuscular disorders, metabolic disorders, cardiopulmonary pathologies, specific musculoskeletal disorders and mental illnesses [27]. Whilst the prevalence of many chronic disease higher in more disadvantaged areas [17], further work is required to determine whether having higher qualified fitness trainers in disadvantaged areas would provide benefits to those communities.

Several approaches can be implemented to address the inequalities observed in this paper. First, government health departments may provide financial incentives for more and higher qualified fitness trainers to work in the most disadvantaged areas. Additionally, incentives such as subsidised access to fitness professionals could be provided to inactive individuals within disadvantaged areas. It would be useful to assess if subsided use of trainers in these areas encouraged more trainers to work in these location and encouraged inactive individuals to engage in exercise. Further, the building of fitness facilities for professionals to work from and that cater to people’s needs is important [14]. Some may not feel comfortable in large community fitness centres, and may favour outdoor or small group settings or vice versa. Our results indicate group training may be more likely in disadvantaged areas (perhaps to share the cost amongst participants) and it is important to ensure these areas have adequate facilities to accommodate group training.

Engagement with fitness trainers and the fitness industry will not be suitable or desired by all individuals. However, given the substantial benefits associated with increasing physical activity levels among the least active [30], the ‘downstream’ public health consequences of providing support to promote physical activity (e.g. reduction in the burden of chronic disease and improvements in quality of life) among populations from disadvantaged areas are likely to be considerable. The pioneering Finnish Diabetes Prevention Study is an example where fitness trainers were effectively utilised in community health promotion [31]. The success of that comprehensive intervention in preventing diabetes among a ‘high-risk’ population was partly credited to the fact that participants were given with free access to community centres and fitness trainers who prescribed individualised exercise programs [31].

### Limitations and strengths

This study has several limitations. First when compared to the most recent demographic data on fitness trainers [20, 24], we appear to have recruited greater proportions of female and older trainers (Additional file 1: Table S1). Therefore, there are restrictions on the generalisability of the findings. It is possible that male and younger trainers work in more disadvantaged areas, thus potentially leading to an overestimation of proportions working in less disadvantaged areas. It is also possible that fitness professionals may work in more than one setting. For practicality, we chose to have trainers report the postcode of setting in which they usually work. Future studies should include an option to report more than one postcode. Finally, we are not able to assess in this study the community benefits of having additional or more highly qualified fitness trainers working in an area. Despite these limitations, our study of the training location of fitness professional contributes to the body of evidence on factors that may be associated with inequalities in physical activity levels. We are not aware of a comparable study that has sampled such a large number of fitness trainers.

## Conclusions

This study showed that a lower proportion of fitness trainers utilises areas with higher levels of socioeconomic disadvantage for their usual training location and that higher fitness industry qualifications was associated with working in areas with lower levels of disadvantage. Future efforts should be made to ensure a greater proportion of and more higher qualified fitness trainers work in disadvantaged areas. Potential strategies may include the provision of incentives for fitness trainers to work in disadvantaged areas and ensuring neighbourhood environments have areas that can be utilised by fitness trainers.

## Additional file

Additional file 1: Representativeness of sample. Data comparing survey responses to existing comparison data on Australian fitness trainers’ sociodemographic characteristics to determine representativeness of the sample. (DOCX 16 kb)
