# Supplementary material for: Variations in area-level disadvantage of Australian registered fitness trainers usual training locations
Source: BMC Public Health. 2016 Jul 11;16:551. doi: 10.1186/s12889-016-3250-3 (PMC4940718; doi:10.1186/s12889-016-3250-3)
Supplement: Additional file 1: — Representativeness of sample. Data comparing survey responses to existing comparison data on Australian fitness trainers’ sociodemographic characteristics to determine representativeness of the sample. (DOCX 16 kb) [file 12889_2016_3250_MOESM1_ESM.docx]

| **Representativeness of sample:**  data comparing survey responses to existing comparison data on Australian fitness trainers’ sociodemographic characteristics to determine representativeness of the sample. | | | | |
| --- | --- | --- | --- | --- |
|  | | **Survey responses^1^** | **Comparison data^2,3^** |  |
|  | |  |  | **% sampled** |
| **Total number** | | n=1,189 | n=29,875^2^ | 3.97 |
| **Sex** | | **%** | **%^3^** | **Diff %** |
|  | Males | 29.0 | 41.0 | -12 |
|  | Females | 71.0 | 59.0 | +12 |
| **Age** | | **%** | **%^2^** | **Diff %** |
|  | 16-17 | 0.2 | 0.2 | 0.0 |
|  | 18-21 | 4.1 | 9.4 | -5.3 |
|  | 22-25 | 8.5 | 15.5 | -7.0 |
|  | 26-29 | 10.5 | 15.3 | -4.8 |
|  | 30-34 | 13.8 | 16.8 | -3.0 |
|  | 35-39 | 13.7 | 14.0 | -0.3 |
|  | 40-44 | 15.6 | 12.0 | +3.6 |
|  | 45-49 | 12.5 | 7.8 | +4.7 |
|  | 50-54 | 10.0 | 5.0 | +5.0 |
|  | 55-59 | 6.1 | 2.4 | +3.7 |
|  | 60-64 | 3.2 | 1.1 | +2.1 |
|  | 65-69 | 0.9 | 0.4 | +0.5 |
|  | 70-83 | 0.3 | 0.1 | +0.2 |
| **State or Territory** | | **%** | **%^2^** | **Diff %** |
|  | NSW/ACT | 36.6 | 38.0 | -1.4 |
|  | Victoria | 23.6 | 19.0 | 4.6 |
|  | Queensland | 19.1 | 23.0 | -3.9 |
|  | South Australia | 6.2 | 6.0 | 0.2 |
|  | Western Australia | 10.8 | 9.5 | 1.3 |
|  | Tasmania | 2.5 | 2.5 | 0 |
|  | Northern Territory | 1.2 | 2.0 | -0.8 |
| **Fitness industry qualification** | | **%** | **%^2^** | **Diff %** |
|  | Certificate III in Fitness | 13.0 | 17.0 | -4.0 |
|  | Certificate IV in Fitness | 68.0 | 77.0 | -9.0 |
|  | Diploma of Fitness | 9.1 | 2.0 | +7.1 |
|  | Tertiary or post-graduate | 9.3 | 3.0 | +6.3 |
| ^1^Current study  ^2^Fitness Industry Workforce Report: https://fitness.org.au/workforcereport.html  ^3^Australian Bureau of Statistics (ABS) Employment in Sport and Recreation, Australia. http://www.abs.gov.au/websitedbs/c311215.nsf/web/Sport+and+Physical+Recreation | | | | |
